# Serum cystatin C is associated with large cerebral artery stenosis in acute ischemic stroke

**DOI:** 10.18632/oncotarget.18061

**Published:** 2017-05-22

**Authors:** Zhiqiang Xu, Cuihua Leng, Bo Yang, Haili Wang, Jing Sun, Zhaoxia Liu, Lingli Yang, Wei Ge, Jiangtao Zhu

**Affiliations:** ^1^ Department of Neurology, The Second Affiliated Hospital of Soochow University, Suzhou City, Jiangsu, China; ^2^ Department of Neurology, The Affiliated Hospital of Xuzhou Medical University, Xuzhou City, Jiangsu, China; ^3^ Department of Radiology, The Second Affiliated Hospital of Soochow University, Suzhou City, Jiangsu, China

**Keywords:** acute ischemic stroke, large cerebral artery stenosis, cystatin C, risk factors

## Abstract

Large cerebral artery stenosis is a major cause of acute ischemic stroke (AIS); however, the correlation between serum cystatin C (CysC) and the stenosis of large cerebral arteries in patients with AIS has not been established. We performed a retrospective review of acute ischemic stroke patients, who were examined by cerebral digital subtraction angiography(DSA). Participants (252 cases) included 131 patients without stenosis and 121 patients with large cerebral artery stenosis. Serum CysC levels in patients with large cerebral artery stenosis were much higher than that of control subjects (p<0.001). However, serum CysC levels were not related to the location of stenosis. Further, logistic regression analyses showed that increased serum CysC was an independent risk factor of large cerebral artery stenosis in patients with acute ischemic stroke. Total participants were subdivided into quintiles based on serum CysC levels. Compared with the first quintile, the odds ratios of risk for large cerebral artery stenosis in the fourth and the fifth quintile were 1.26 (p<0.05) and 4.71(p<0.05) respectively, after the adjustment for age, sex, and smoking, hypertension, type 2 diabetes mellitus(DM), dyslipidemia, creatinine(Cr), urea, uric acid, and C reactive protein(CRP). Therefore, a significant positive correlation was observed between elevated serum CysC levels and large cerebral artery stenosis in patients with acute ischemic stroke. In summary, our findings provide new insights into the correlation between increased serum CysC and large cerebral artery stenosis in patients with acute ischemic stroke.

## INTRODUCTION

Acute ischemic stroke contributes substantially to the global burden of disease and disability with the aging of the population [1, 2]. The cost of stroke and Stroke cytoprotection were high, with a global stroke incidence of 10.3–16.9 million annually and a high risk for recurrence in patients who suffer an acute stroke [3, 4]. Large-artery atherosclerosis subtype is one of the main types of acute ischemic stroke. The cumulative 1-year event rates of noncardioembolic ischemic stroke were 18% for combined vascular events in China [5]. Hypertension, hyperlipidemia and diabetes are involved in the development of atherosclerosis,which plays an important role in the process of large cerebral artery stenosis. Patients with well-controlled these factors still have a high incidence of cardiovascular events [6–8]. Thus, finding the risk factors of large cerebral artery stenosis is a topic of considerable interest.

Cystatin C (CysC), a 13-kD protein, is encoded by CST3 gene and constitutively expresses in all nucleated cells [9]. CysC is considered as an excellent biomarker of glomerular filtration rate estimation [10] because serum CysC is better than serum creatinine in the evaluation of renal function [10, 11]. Moreover, Serum CysC levels are less dependent on muscle mass, age, race and sex than creatinine [12]. In addition, serum CysC also serves as an indicator of renal function for the adjustment of medication doses [13, 14]. A recent study demonstrated a close correlation between elevated serum CysC levels and ischemic stroke or hemorrhagic stroke, and increased serum CysC may be used as a predictor for high risk of stroke or death [15]. Our previous studies also demonstrated that increased serum CysC is an independent risk factor for acute ischemic stroke [16]. Thus, it is important to detect the levels of serum CysC in patients with stroke.

CysC, an excellent biomarker of glomerular filtration rate estimation, is a risk factor for cardiovascular events [17]. Patients with carotid atherosclerosis have higher concentration of serum CysC [18, 19]. However, the correlation between increased serum CysC and the stenosis of large cerebral arteries in patients with AIS is unclear. Therefore, we examined the relationship between CysC and large cerebral artery stenosis in patients with acute ischemic stroke.

## RESULTS

### Serum CysC increased in patients with AIS and stenosis

Among 252 patients with AIS, 131 subjects (52.0%) were categorized as the normal group without large cerebral artery stenosis and 121 patients (48.0%) were in stenosis group with large cerebral artery stenosis. Demographics, baseline physical exam characteristics, and laboratory variables were listed in Table 1. There was no difference in age, sex, SBP, and CRP between control group and stenosis group.

**Table 1:** Demographic characteristics of patients with ischemic stroke

Characteristics	Normal(n=131)	Stenosis(n=121)	P*
Age (years ^a^)	61 (19.0)	65 (17.0)	0.20
Sex (M/W)	102/29	96/25	0.78
Smoking, n (%)	53(40.5)	48(39.7)	0.90
Hypertension, n (%)	89 (67.9)	83 (68.6)	0.91
DM, n (%)	22 (16.8)	15 (12.4)	0.32
Dyslipidemia, n (%)	24(18.3)	25(20.7)	0.64
SBP (mmHg ^b^)	145± 19.6	147± 21.0	0.562
DBP (mmHg ^a^)	80 (18)	84 (78, 93)	0.45
FBG (mmol/L^a^)	5.44 (1.59)	5.47(1.35)	0.419
Urea (mmol/L^a^)	4.60 (1.90)	4.70 (1.70)	0.48
CRP(mmol/L ^a^)	5.60 (2.20)	5.60(2.90)	0.35
Cr (lmol/L ^a^)	65.0(18.0)	70.0(27.0)	0.011
Uric acid (mmol/L ^b^)	315 ± 88.7	323 ± 89.4	0.46
Calcium	2.19(0.17)	2.23(0.19)	0.034
Hcy(mmol/L ^a^)	16.67 (5.17)	16.67 (4.92)	0.929
Circulation, n (%)			
Anterior	72(55.0)	61(50.4)	
Posterior	24(18.3)	18(14.9)	0.37
Both	35(26.7)	42(34.7)	
Stroke classification by TOAST criteria, n (%)			
Large-artery atherosclerosis	0 (0)	66(54.5)	
Cardioembolism	21(16.0)	6(5.0)	
Small-vessel occlusion	60(45.8)	31(25.6)	<0.001
Other etiology	4(3)	4(3.3)	
Undetermined etiology	46(35.1)	14(11.6)	

A →* p value<0.05 was considered statistically significant. n: number; M: men; W: women; DM: diabetes; SBP: systolic blood pressure; DBP: diastolic blood pressure; FBG: fasting blood glucose; Cr: creatinine; CRP: C-reactive protein; SD: standard deviation; IQR: interquartile range.

^a^ Data represented the median(IQR).

^b^ Data represented mean ± SD.

**Figure 1:**
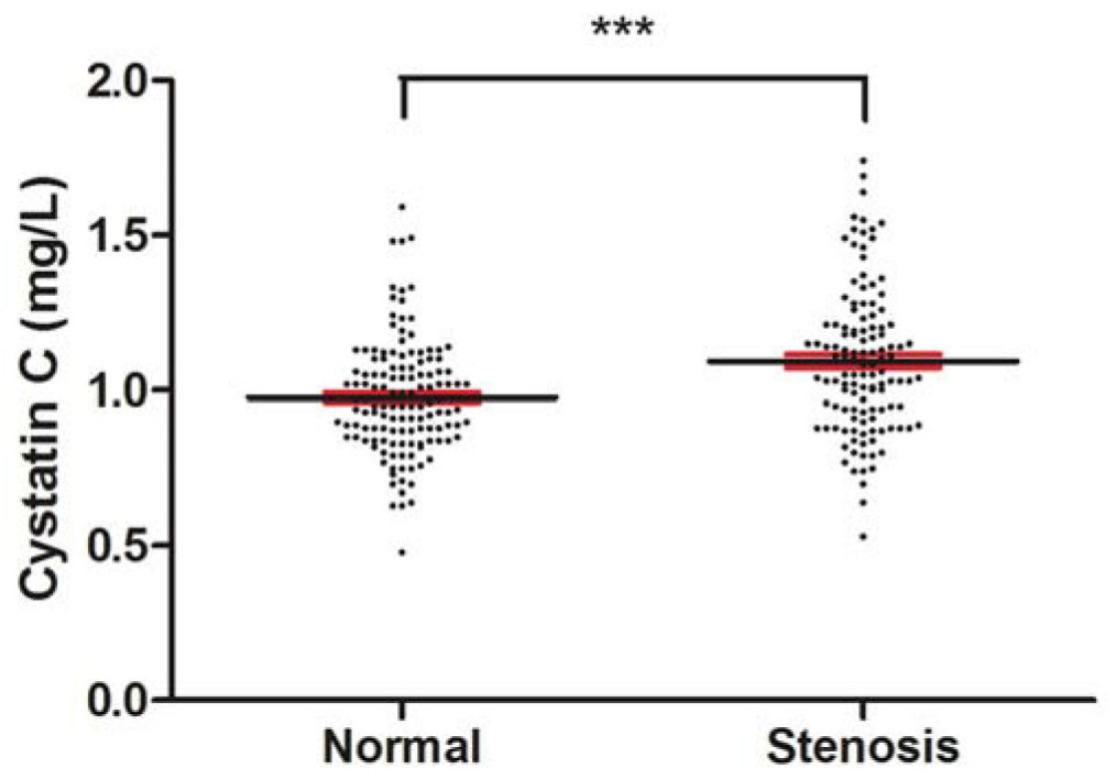
Serum concentration of Cystatin C (mean±2SE) Serum cystatin C was significantly elevated in the patients with artery stenosis. Serum CysC levels were measured after overnight fasting at the second day after admission by using venous blood samples. (***p value<0.001, versus normal group).

Among the patients, serum CysC levels increased significantly in stenosis group compared with normal group (P<0.001) as shown in Figure 1. These data suggested that the increase of serum CysC was related to large cerebral artery stenosis in patients with AIS, even when those possible confounders (age, sex, smoking, type 2 DM, dyslipidemia, Cr, Urea, Uric acid, hcy, CRP) were adjusted as shown in Table 2.

**Table 2:** Adjusted mean (±SD) values of serum concentration of CysC

Serum CysC	Adjusted CysC(mg/L)		P*
	Normal	Stenosis	
Crude	0.98 ± 0.21	1.09 ± 0.21	< 0.001
Model 1	0.98 ±0.18	1.09 ± 0.19	<0.001
Model 2	0.99 ± 0.18	1.08 ±0.19	<0.001
Model 3	1.00 ± 0.15	1.06 ±0.15	0.002
Model 4	1.00 ± 0.15	1.06 ±0.15	0.003

Model 1 included age, sex, and smoking; Model 2 included variables in model 1 plus hypertension, type 2 DM, Dyslipidemia; Model 3 included variables in model 2 plus values of Cr, Urea, Uric acid; Model 4 included variables in model 3 plus values of hcy, CRP. *p values was generated by general linear models, with adjustment for pairwise comparisons.

### Serum CysC was highly associated with large cerebral artery stenosis but not the location of large cerebral artery stenosis in patients with AIS

To examine the relationship between increased serum CysC and large cerebral artery stenosis, regression analyses were performed in patients with AIS. The results indicated that CysC was a risk factor for large cerebral artery stenosis in patients with AIS(OR=15.35, 95 %CI 4.17–56.54, p < 0.001; Table 3). After adjusting according to potential confounders, such as age, sex, and smoking, hypertension, type 2 DM, dyslipidemia, hcy, and CRP, CysC is an independent risk factor for large cerebral artery stenosis in patients with AIS (OR=17.46, 95 %CI 2.70−113.14, p=0.003; Table 3).

**Table 3:** Logistic regression analysis for large cerebral artery stenosis

Serum CysC	Stenosis		P*
	OR	95% confidence intervals	
Crude	15.35	4.17-56.54	<0.001
Model 1	19.70	4.60-84.28	<0.001
Model 2	19.32	4.45-83.92	<0.001
Model 3	16.74	2.67-105.09	0.003
Model 4	17.46	2.70-113.14	0.003

Variables and conditions of the models are the same as in Table 2.

As showed in Table 4, patients with large cerebral artery stenosis were divided into 4 groups, vertebral-basilar artery stenosis group (VBA stenosis), internal carotid artery stenosis group(ICA stenosis), middle cerebral artery stenosis group (MCA stenosis), and at least 2 large cerebral artery stenosis group (Combined stenosis). General linear model analysis indicated that there were no significant differences for serum CysC among the groups even after adjustment according to the confounders such as age, sex, and smoking, hypertension, type 2 DM, dyslipidemia, hcy, and CRP. These data suggested that serum CysC were not associated with the location of large cerebral artery stenosis in patients with AIS.

**Table 4:** Logistic regression analysis for the position of large cerebral artery stenosis

Serum CysC	Adjusted CysC(mg/L)				P*
	VBA stenosis	CIA stenosis	MCA stenosis	Combined stenosis	
Crude	1.03 ±0.50	1.10 ±0.41	1.14 ±0.46	1.10 ±0.51	0.340
Model 1	1.05 ±0.42	1.09 ±0.34	1.11 ±0.40	1.12 ±0.43	0.629
Model 2	1.05 ±0.43	1.09 ±0.34	1.11 ±0.40	1.12 ±0.44	0.570
Model 3	1.06 ±0.35	1.08 ±0.28	1.09 ±0.32	1.15 ±0.36	0.255
Model 4	1.06 ±0.35	1.09 ±0.29	1.09 ±0.33	1.15 ±0.36	0.301

Variables and conditions of the models are the same as in Table 2.

### Patients with high serum CysC had a high risk of large cerebral artery stenosis

As previously reported [16], total patients were divided into five subsets by using cutoff points at the 20th, 40th, 60th, and 80th percentiles of serum CysC values. After adjustment according to potential confounders such as age, sex, and smoking, hypertension, type 2 DM, dyslipidemia, hcy, and CRP, the odds ratios (and 95 %CIs) for large cerebral artery stenosis were analyzed (Table 5). Compared with the first quintile, the fourth and the fifth quintile of the odds ratios (95% confidence intervals) for large cerebral artery stenosis were 1.26(1.03-6.77, p<0.05) and 4.71(1.45-15.26, p<0.05) after adjustment for age, sex, and smoking, hypertension, type 2 DM, Dyslipidemia, Cr, Urea, Uric acid, hcy, CRP. Patients with low serum CysC in quintile 1 to 3 demonstrated a similar risk of having a large cerebral artery stenosis, while patients with high serum CysC in quintile 4 (P<0.05, Table 5) and quintile 5 (P<0.05, Table 5) were at high risk of having large cerebral artery stenosis.

**Table 5:** The association between CysC and the risk of stenosis

Serum CysC	Quintile 1	Quintile 2	Quintile 3	Quintile 4	Quintile 5
Range of values (mg/L)	<0.86	0.87–0.95	0.96–1.06	1.07–1.20	>1.21
No. of cases	50	55	52	51	44
No. of stenosis	16	22	23	29	31
Incidence, %	34.2	34.6	46.9	39.6	73.8
Odds ratio (95 % confidence interval)					
Unadjusted	1.00	1.42(0.64-3.16)	1.68(0.75-3.78)	2.80(1.24-6.31)*	5.07(2.10-12.20)***
Adjusted	1.00	1.36(0.59-3.15)	1.51(0.61-3.71)	1.26(1.03-6.77)*	4.71(1.45-15.26)*

Variables and conditions included age, sex, and smoking, hypertension, type 2 DM, Dyslipidemia, Cr, Urea, Uric acid, hcy, CRP. (*p value <0.05, *** p value <0.001)

## DISCUSSION

The incidence of AIS is rapidly increasing with associated the aging of the population [1, 2]. The independent risk factors of ischemic heart disease had highly improved treatment strategy on myocardial infarctio [20], meanwhile, efficient blood independent risk factors of large cerebral artery stenosis are urgently required [21]. The present study showed markedly elevated serum CysC levels in AIS patients with large cerebral artery stenosis compared with patients without large cerebral artery stenosis. More importantly, our data indicated that increased serum CysC was an independent risk factor of large cerebral artery stenosis.

CysC is highly espressed in the central nervous system [22]. Studies reported that the concentration of CysC in cerebrospinal fluid is more than 5 times in the blood, which implies that CysC plays an important role in the central nervous system [23]. The close association between increased serum CysC and cerebral small vessel disease has been reported [24]. In this study, it is the first time to use DSA for the evaluation of large cerebral artery stenosis and examine the relationship between increased serum CysC and large cerebral artery stenosis. So far, DSA remains the golden standard for lumenographic imaging method and visualizing the degree of stenosis with excellent resolution, despite of the fact that it has some disadvantages [25, 26]. Our findings demonstrated that increased serum CysC was an independent risk factor for large cerebral artery stenosis in patients with AIS.

Major mechanisms regulating cerebral artery function have been reported [27], and atherosclerosis may predispose to clinical stroke. Importantly, molecular targets that may be of beneft for patients in clinical stroke and perhaps the prevention or treatment of ischemic stroke, were identifed. Cerebral artery function is very sensitive to endothelial dysfunction that occurs during chronic disease, resulting in impairment of vasodilator mechanisms. Atherosclerosis is the underlying pathological process for large cerebral artery stenosis, which is a main cause of cerebrovascular disease [28].

The presence of renal dysfunction is closely associated with cardiovascular events. A recent study demonstrated that decreased epidermal glomerular filtration rate is only associated with an increased risk of ischemic stroke [29]. Moreover, microalbuminuria associated with vascular endothelial dysfunction in deep small infarcts and cerebral small vessel disease have been reported [29–31]. Recent study showed that increased CysC levels were more closely related to large-artery atherosclerosis (LAA) subtype of cerebral infarction than other subtype [32], suggesting CysC may play a role in the process of atherosclerosis. The elevated levels of CysC in serum were not just in acute stress reaction [33]. Chronic inflammation is known to promote the development of atherosclerosis [34], The correlation of CysC with chronic inflammation was discussed [35], which implied CysC may be involved in the process of large cerebral artery stenosis via chronic inflammation. In addition, as an inhibitor of cysteine proteases, high levels of serum CysC directly affect the process of vascular wall remodeling by breaking the balance of proteolytic and anti-proteolytic activities [36]. Importantly, our data reported that elevated CysC levels were an independent risk factor for large cerebral artery stenosis in patients with AIS. Thus, it is important to detect the levels of CysC, especially in AIS patients with large cerebral artery stenosis.

There are some limitations in this study. Firstly, data were gathered from a single hospital and sample size was limited: this may caused inherent biases. Secondly, We have only examined the levels of CysC during hospitalization, it could not reveal whether the increased CysC levels were an outcome or a causative factor in cerebral vessel disease. Thirdly, the mechanism of independent association of increased the serum CysC with large artery stenosis remains unclear, and further experiments to explain are needed. Therefore, further follow-up study is needed. In addition, the relationship between cerebral vessel stenosis and serum CysC levels still needs to be elucidated by careful design of longitudinal studies.

In summary, although further validation and evaluation in clinical settings are needed, the present findings showed that increased serum CysC was highly associated with large cerebral artery stenosis and was an independent risk factor of large cerebral artery stenosis in patients with AIS.

## MATERIALS AND METHODS

### Patients

This study was approved by the Ethics Committee of the Second Affiliated Hospital of Soochow University. Total 252 patients with acute ischemic stroke who were examined with cerebral digital subtraction angiography (DSA) within 7 days stroke from January 2012 to December 2015 in the Second Affiliated Hospital of Soochow University were enrolled in this study. All the patients provided informed consent forms to participate. Acute ischemic stroke were defined as acute focal neurological deficits, which lasted more than 24 hours and was confirmed by the presence of computed tomography and/or magnetic resonance imaging. Large cerebral artery stenosis was defined as a narrowing of the relevant artery lumen of ≥50% or occlusion by viewing cerebral DSA videos. Accordingly, all patients were divided into two groups: stenosis group and normal group. Ischemic strokes were categorized originally into five groups as recommended by the diagnostic criteria [37]: large-artery atherosclerosis, cardioembolism, small-vessel occlusion, other etiology, and undetermined etiology. The exclusion criteria included patients with encephalitis, neurodegenerative diseases, hematological disorders, and serious systemic diseases such as urinary, cancer, trauma, infectious.

### Measurement of serum CysC and other risk factors

Serum CysC, urea, creatinine, C-reactive protein (CRP), uric acid, triglycerides, high-density lipoprotein (HDL) cholesterol, fasting plasma glucose, low-density lipoprotein (LDL) cholesterol, and total cholesterol (TC) were measured after overnight fasting by using venous blood samples. The methods for these biochemical parameters were performed as in our previously reported [16]. Hypertension was considered to be present if patients had been previously diagnosed according to guidelines set by the World Health Organization and the International Society of Hypertension. Diabetes mellitus was determined by the previous use of anti-diabetic medication, fasting blood glucose ≥ 7.0 mM, or postprandial blood glucose after 2 hours ≥11.1 mM.

### Cerebral DSA examination

Large cerebral artery was defined as the internal carotid artery main stem, the middle cerebral artery (M1), and the vertebral-basilar artery [38, 39]. Large cerebral artery stenosis was defined as a narrowing of the relevant artery lumen of ≥50% or occlusion by viewing cerebral DSA videos [40]. Angiography was performed through a common femoral artery approach, using a standard guidewire and a 5-French catheter. DSA images were observed on a Philips biplane Easy Vision workstation with anterior-posterior, oblique and lateral views, utilizing a 1024 × 1024 matrix and a 30-cm-diameter image intensifier. Two observers assessed luminal narrowing of cerebral vessel disease by viewing DSA videos without knowing about the symptomatic side.

### Statistical analysis

Descriptive statistics for continuous variables were expressed as the mean ± standard deviation (SD); categorical variables were expressed as constituent ratios. For serum CysC, mean values (±2SE) were depicted graphically based on the cerebral artery status. A general linear model was used to estimate the relationship between adjusted CysC values and large cerebral artery stenosis. Bonferroni tests were implemented to correct the error generated by multiple comparisons. Finally, the association between large cerebral artery stenosis and CysC levels were estimated with regression analyses, and the odds ratio (OR) and 95% confidence intervals (CI) were estimated. All statistical analyses were performed by using the SPSS software (version 24.0) (SPSS Inc., Chicago, IL, USA).

## References

[1] Feigin VL (2005). Stroke epidemiology in the developing world. Lancet.

[2] Pennypacker KR, Bix G, Fraser JF (2017). Correcting the trajectory of stroke therapeutic research. Transl Stroke Res.

[3] Lapchak PA, Zhang JH (2017). The high cost of stroke and stroke cytoprotection research. Transl Stroke Res.

[4] Imam YZ, D'Souza A, Malik RA, Shuaib A (2016). Secondary stroke prevention: improving diagnosis and management with newer technologies. Transl Stroke Res.

[5] Xu H, Ping Y, Lin H, He P, Li W, Dai H (2017). Antiplatelet strategies and outcomes in patients with noncardioembolic ischemic stroke from a real-world study with a five-year follow-up. Transl Stroke Res.

[6] UK Prospective Diabetes Study (UKPDS) Group (1998). Intensive blood-glucose control with sulphonylureas or insulin compared with conventional treatment and risk of complications in patients with type 2 diabetes (UKPDS 33). Lancet.

[7] He J, Whelton PK (1999). Elevated systolic blood pressure and risk of cardiovascular and renal disease: overview of evidence from observational epidemiologic studies and randomized controlled trials. Am Heart J.

[8] Saito Y, Shirai K, Sasaki N, Shinomiya M, Yoshida S, Committee of the Chiba Lipid Intervention Program Study (2002). Prognosis of hypercholesterolemic patients taking pravastatin for five years: the Chiba Lipid Intervention Program (CLIP) Study. J Atheroscler Thromb.

[9] Abrahamson M, Olafsson I, Palsdottir A, Ulvsback M, Lundwall A, Jensson O, Grubb A (1990). Structure and expression of the human cystatin C gene. Biochem J.

[10] Dharnidharka VR, Kwon C, Stevens G (2002). Serum cystatin C is superior to serum creatinine as a marker of kidney function: a meta-analysis. Am J Kidney Dis.

[11] Roos JF, Doust J, Tett SE, Kirkpatrick CM (2007). Diagnostic accuracy of cystatin C compared to serum creatinine for the estimation of renal dysfunction in adults and children--a meta-analysis. Clin Biochem.

[12] Stevens LA, Coresh J, Schmid CH, Feldman HI, Froissart M, Kusek J, Rossert J, Van Lente F, Bruce RD, Zhang YL, Greene T, Levey AS (2008). Estimating GFR using serum cystatin C alone and in combination with serum creatinine: a pooled analysis of 3,418 individuals with CKD. Am J Kidney Dis.

[13] Hermida J, Tutor JC (2006). Serum cystatin C for the prediction of glomerular filtration rate with regard to the dose adjustment of amikacin, gentamicin, tobramycin, and vancomycin. Ther Drug Monit.

[14] Schuck O, Teplan V, Sibova J, Stollova M (2004). Predicting the glomerular filtration rate from serum creatinine, serum cystatin C and the Cockcroft and Gault formula with regard to drug dosage adjustment. Int J Clin Pharmacol Ther.

[15] Ni L, Lu J, Hou LB, Yan JT, Fan Q, Hui R, Cianflone K, Wang W, Wang DW (2007). Cystatin C, associated with hemorrhagic and ischemic stroke, is a strong predictor of the risk of cardiovascular events and death in Chinese. Stroke.

[16] Yang B, Zhu J, Miao Z, Zhou B, Ge W, Zhao H, Xu X (2015). Cystatin C is an independent risk factor and therapeutic target for acute ischemic stroke. Neurotox Res.

[17] Hoke M, Amighi J, Mlekusch W, Schlager O, Exner M, Sabeti S, Dick P, Koppensteiner R, Minar E, Rumpold H, Wagner O, Schillinger M (2010). Cystatin C and the risk for cardiovascular events in patients with asymptomatic carotid atherosclerosis. Stroke.

[18] Tejera-Segura B, de Vera-Gonzalez AM, Lopez-Mejias R, Gonzalez-Gay MA, Ferraz-Amaro I (2016). Serum cathepsin S and cystatin C: relationship to subclinical carotid atherosclerosis in rheumatoid arthritis. Clin Exp Rheumatol.

[19] Umemura T, Kawamura T, Mashita S, Kameyama T, Sobue G (2016). Higher levels of cystatin C are associated with extracranial carotid artery steno-occlusive disease in patients with noncardioembolic ischemic stroke. Cerebrovasc Dis Extra.

[20] Vasan RS (2006). Biomarkers of cardiovascular disease: molecular basis and practical considerations. Circulation.

[21] Kim JM, Jung KH, Chu K, Lee ST, Ban J, Moon J, Kim M, Lee SK, Roh JK (2015). Atherosclerosis-related circulating microRNAs as a predictor of stroke recurrence. Transl Stroke Res.

[22] Hakansson K, Huh C, Grubb A, Karlsson S, Abrahamson M (1996). Mouse and rat cystatin C: Escherichia coli production, characterization and tissue distribution. Comp Biochem Physiol B Biochem Mol Biol.

[23] Mathews PM, Levy E (2016). Cystatin C in aging and in Alzheimer's disease. Ageing Res Rev.

[24] Wada M, Nagasawa H, Kawanami T, Kurita K, Daimon M, Kubota I, Kayama T, Kato T (2010). Cystatin C as an index of cerebral small vessel disease: results of a cross-sectional study in community-based Japanese elderly. Eur J Neurol.

[25] Liu Q, Huang J, Degnan AJ, Chen S, Gillard JH, Teng Z, Lu J (2013). Comparison of high-resolution MRI with CT angiography and digital subtraction angiography for the evaluation of middle cerebral artery atherosclerotic steno-occlusive disease. Int J Cardiovasc Imaging.

[26] Villablanca JP, Rodriguez FJ, Stockman T, Dahliwal S, Omura M, Hazany S, Sayre J (2007). MDCT angiography for detection and quantification of small intracranial arteries: comparison with conventional catheter angiography. AJR Am J Roentgenol.

[27] Ross R (1995). Cell biology of atherosclerosis. Annu Rev Physiol.

[28] Hansson GK, Hermansson A (2011). The immune system in atherosclerosis. Nat Immunol.

[29] Mahmoodi BK, Yatsuya H, Matsushita K, Sang Y, Gottesman RF, Astor BC, Woodward M, Longstreth WT, Psaty BM, Shlipak MG, Folsom AR, Gansevoort RT, Coresh J (2014). Association of kidney disease measures with ischemic versus hemorrhagic strokes: pooled analyses of 4 prospective community-based cohorts. Stroke.

[30] Umemura T, Senda J, Fukami Y, Mashita S, Kawamura T, Sakakibara T, Sobue G (2014). Impact of albuminuria on early neurological deterioration and lesion volume expansion in lenticulostriate small infarcts. Stroke.

[31] Umemura T, Kawamura T, Sakakibara T, Mashita S, Hotta N, Sobue G (2012). Microalbuminuria is independently associated with deep or infratentorial brain microbleeds in hypertensive adults. Am J Hypertens.

[32] Zeng Q, Lin K, Yao M, Wei L (2015). Significant correlation between cystatin C, cerebral infarction, and potential biomarker for increased risk of stroke. Curr Neurovasc Res.

[33] Xiao D, Liu H, Zhang H, Luo Y (2012). Impact of cystatin C levels on infarct size and hemorrhage volume in acute cerebral stroke. J Neurol.

[34] Kitano T, Nezu T, Shiromoto T, Kubo S, Uemura J, Wada Y, Yagita Y (2017). Association between absolute eosinophil count and complex aortic arch plaque in patients with acute ischemic stroke. Stroke.

[35] Salgado JV, Souza FL, Salgado BJ (2013). How to understand the association between cystatin C levels and cardiovascular disease: imbalance, counterbalance, or consequence?. J Cardiol.

[36] Liu J, Sukhova GK, Sun JS, Xu WH, Libby P, Shi GP (2004). Lysosomal cysteine proteases in atherosclerosis. Arterioscler Thromb Vasc Biol.

[37] Adams HP, Bendixen BH, Kappelle LJ, Biller J, Love BB, Gordon DL, Marsh EE (1993). Classification of subtype of acute ischemic stroke. Definitions for use in a multicenter clinical trial. TOAST. Trial of Org 10172 in Acute Stroke Treatment. Stroke.

[38] Zhao L, Barlinn K, Sharma VK, Tsivgoulis G, Cava LF, Vasdekis SN, Teoh HL, Triantafyllou N, Chan BP, Sharma A, Voumvourakis K, Stamboulis E, Saqqur M (2011). Velocity criteria for intracranial stenosis revisited: an international multicenter study of transcranial Doppler and digital subtraction angiography. Stroke.

[39] Heldner MR, Zubler C, Mattle HP, Schroth G, Weck A, Mono ML, Gralla J, Jung S, El-Koussy M, Ludi R, Yan X, Arnold M, Ozdoba C (2013). National Institutes of Health stroke scale score and vessel occlusion in 2152 patients with acute ischemic stroke. Stroke.

[40] Liu J, Zhang N, Fan Z, Luo N, Zhao Y, Bi X, An J, Chen Z, Liu D, Wen Z, Fan Z, Li D (2016). Image quality and stenosis assessment of non-contrast-enhanced 3-T magnetic resonance angiography in patients with peripheral artery disease compared with contrast-enhanced magnetic resonance angiography and digital subtraction angiography. PLoS One.

